# Therapeutic effects of hepatocyte growth factor-overexpressing dental pulp stem cells on liver cirrhosis in a rat model

**DOI:** 10.1038/s41598-017-14995-5

**Published:** 2017-11-17

**Authors:** Xiao-fang Cao, Shi-zhu Jin, Liang Sun, Yuan-bo Zhan, Feng Lin, Ying Li, Ying-lian Zhou, Xiu-mei Wang, Li Gao, Bin Zhang

**Affiliations:** 1 Department of Dentistry, Second Affiliated Hospital of Harbin Medical University, Harbin, 150086 Heilongjiang China; 2 Department of Gastrointestinal and Hepatology, Second Affiliated Hospital of Harbin Medical University, Harbin, 150086 Heilongjiang China; 3 Department of Human Anatomy, Harbin Medical University, Harbin, 150081 Heilongjiang China; 4 Institute of Hard Tissue Development and Regeneration, Second Affiliated Hospital of Harbin Medical University, Harbin, 150001 Heilongjiang China; 5 Department of neurology, The Second Affiliated Hospital of Harbin Medical University, Harbin, 150086 Heilongjiang China; 6 Heilongjiang Academy of Medical Sciences, Harbin, 150001 Heilongjiang China

## Abstract

Cirrhosis is the terminal stage of hepatic diseases and is prone to develop into hepatocyte carcinoma. Increasing evidence suggests that the transplantation of dental pulp stem cells (DPSCs) may promote recovery from cirrhosis, but the key regulatory mechanisms involved remain to be determined. In this study, we overexpressed human hepatocyte growth factor (hHGF) in primary rat DPSCs and evaluated the effects of HGF overexpression on the biological behaviors and therapeutic efficacy of grafted DPSCs in cirrhosis. Liver cirrhosis was induced via the intraperitoneal injection of CCl_4_ twice weekly for 12 weeks and was verified through histopathological and serological assays. HGF was overexpressed in DPSCs via transduction with a hHGF-lentiviral vector and confirmed based on the elevated expression and secretion of HGF. The HGF-overexpressing DPSCs were transplanted into rats intravenously. The HGF-overexpressing DPSCs showed increased survival and hepatogenic differentiation in host liver tissue at 6 weeks after grafting. They also exhibited a significantly greater repair potential in relation to cirrhosis pathology and impaired liver function than did DPSCs expressing HGF at physiological levels. Our study may provide an experimental basis for the development of novel methods for the treatment of liver cirrhosis in clinical practice.

## Introduction

Cirrhosis, characterized by diffuse degeneration and the death of hepatocytes, followed by nodular regeneration, extensive fibrosis and the consequent collapse of the normal liver architecture, is the terminal stage of a variety of chronic liver diseases, resulting in irreversible impaired liver function, portal hypertension and the potential to develop into hepatocellular carcinoma^1,2^. Epidemiological data show that liver cirrhosis is the 14th most common cause of death worldwide but the fourth in central Europe^3^. Because of the lack of effective anti-fibrotic methods currently available in clinical practice^3–5^, liver transplantation remains the only chance of a cure for patients with liver cirrhosis and is severely restricted by the source of donor organs. Hence, alternative therapeutic strategies are needed.

Mesenchymal stem cell (MSC) transplantation, which has shown notable potential for repairing hepatic architecture and function in preclinical studies, may represent a prospective therapy for liver fibrosis^6^. Dental pulp stem cells (DPSCs) are a unique type of dental pulp-enriched MSC that hold promise for use in tissue engineering and regenerative therapies. DPSCs are widely available, proliferate rapidly and thus easily amplified, have preserved multipotency, are able to survive during long-term cryopreservation and have minimal immune rejection when autografted^7–10^. Grafted DPSCs show an effective regenerative capacity in cardiac injury following ischemia-reperfusion as well as central nervous system injury, without teratoma formation^11,12^. Recently, DPSCs have been reported to differentiate into functional hepatocyte-like cells and express hepatic markers *in vitro* and *in vivo* ^13,14^. However, the determinant induction of hepatic differentiation of DPSCs and the therapeutic efficacy of DPSC grafting for cirrhosis remain poorly understood.

Hepatocyte growth factor (HGF), which is mainly secreted by mesenchymal cells, functions as a cell growth, motility, morphogenic and antiapoptotic factor by activating c-Met receptor/tyrosine kinase signaling in many cell types and plays critical roles in embryonic organogenesis, adult organ regeneration and wound healing^15,16^. HGF has also been verified to show anti-fibrotic activity in both the onset and progression of liver fibrosis/cirrhosis^17,18^. However, high exogenous HGF protein levels cannot be maintained, as the protein is extremely unstable (half-life <15 min)^19^. Therefore, the selection of a vehicle for the long-term transfer of therapeutic genes is important. For liver regeneration, MSCs are promising candidate. For example, transplantation of bone marrow mesenchymal stem cells (BMMSCs) and umbilical cord blood-derived mesenchymal stem cells (UCB-MSCs) overexpressing HGF in a rat model of liver fibrosis was shown to ameliorate liver injury induced by the hepatotoxin carbon tetrachloride (CCl_4_)^20,21^. However, the impact of HGF on DPSCs and the therapeutic efficacy of DPSCs in cirrhosis are still elusive.

Therefore, in the present study, we overexpressed human HGF (hHGF) in rat-derived primary DPSCs through the transduction of a hHGF-expressing lentiviral vector and evaluated the effects of elevated HGF expression on the survival, fate determination and regenerative capacity of grafted DPSCs in a rat model of cirrhosis to provide an experimental basis for the development of a novel anti-cirrhosis therapy.

## Materials and Methods

### Study design

**Figure 1:**
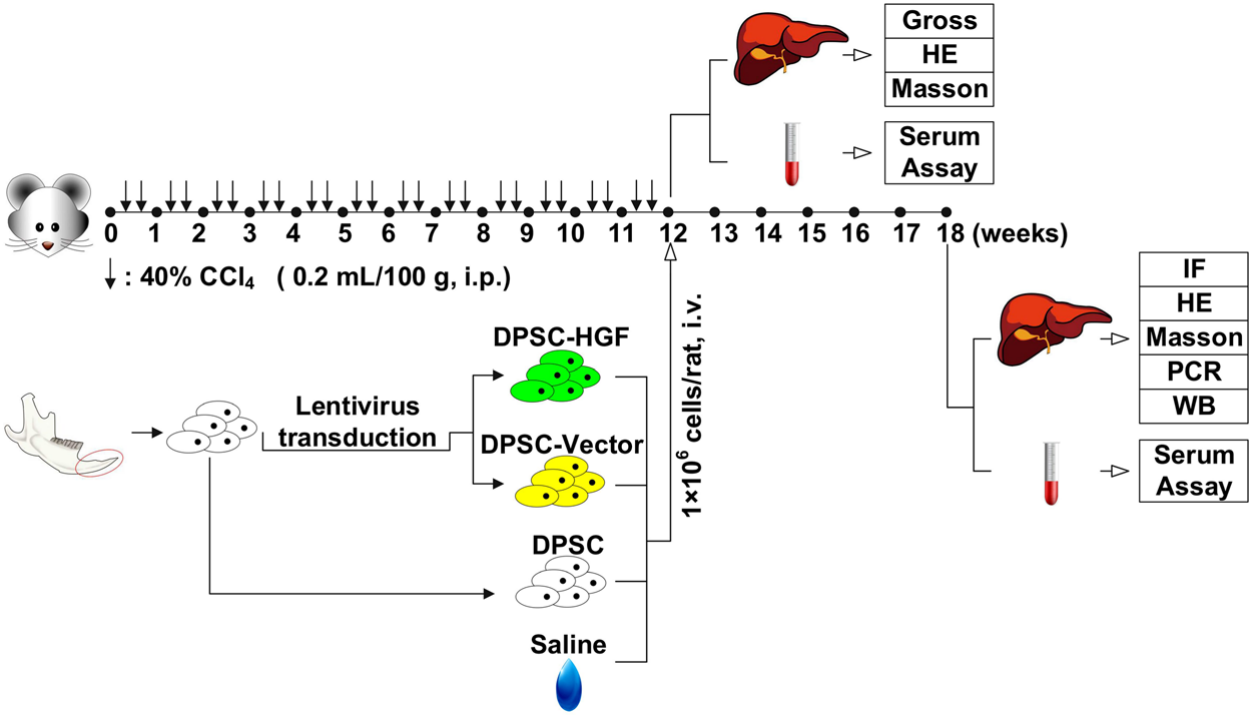
Outline of the experimental design. Science Slides 2005 was used to crate these images (www.scienceslides.com).

All animal experiments were conducted in strict accordance with the principles of medical ethics and were approved by the ethics committee of the Hospital of Harbin Medical University. As shown in Fig. 1, liver cirrhosis was induced in female Sprague-Dawley rats(200–250 g, purchased from the Department of the Animal Experiment Center of the Second Affiliated Hospital of Harbin Medical University) via the intraperitoneal injection of CCl_4_ for 12 weeks and was verified through histopathological and serological analyses. DPSCs were derived from the incisors of 4-week-old Sprague-Dawley rats. To assess their purity and multipotency, DPSCs at the 3^rd^ passage were subjected to fluorescence-activated cell sorting (FACS) to detect surface markers or were differentiated into osteoblasts, adipocytes and hepatocytes. To investigate the impacts of elevated HGF expression on the therapeutic efficacy of DPSC grafting for liver cirrhosis, the experimental animals (50 female Sprague-Dawley rats, 200–250 g) were randomly divided into one of the following 5 groups: Control, CCl_4_/Saline, CCl_4_/DPSC, CCl_4_/DPSC-Vector, or CCl_4_/DPSC-HGF. The rats in the Control group were administered intraperitoneal injections of saline for 12 weeks. The animals in the other groups were administered intraperitoneal CCl_4_ injections for the same period. The rats in the CCl_4_/Saline, CCl_4_/DPSC, CCl_4_/DPSC-Vector and CCl_4_/DPSC-HGF groups underwent treatment with saline alone, wild-type DPSCs, blank lentiviral vector-transduced DPSCs or hHGF-expressing lentiviral vector-transduced DPSCs, respectively, in an equal volume via intravenous injection on Week 12. On Week 18, immunofluorescence (IF) staining was performed to determine the survival and differentiation of the grafted DPSCs in host hepatic tissue. Histopathological and molecular analyses were conducted to evaluate histological restoration, and serum assays were conducted to assess the recovery of liver function.

### Cell culture

DPSCs were isolated from rat incisors as described previously, with some modifications^22^. Briefly, dental pulp was extracted from the lower incisors of 4-week-old male Sprague-Dawley rats, then cut into pieces of less than 1 mm in 0.01 M phosphate-buffered saline (PBS; GIBCO-BRL, USA)^23^ and digested with type I collagenase (3 mg/mL; Sigma-Aldrich, USA) for 45 min (37 °C, 8 rpm). Digestion was neutralized with DPSC complete medium (Dulbecco’s modified Eagle’s medium (DMEM; GIBCO-BRL) containing 10% fetal bovine serum (FBS; GIBCO-BRL), 0.292 mg/mL glutamine (Invitrogen, USA), 100 units/mL penicillin G (GIBCO-BRL), 100 mg/mL streptomycin (GIBCO-BRL), and 2.5 mg/mL ascorbic acid (GIBCO-BRL)). After filtration, the cells were harvested and plated in 25 cm^2^ culture flasks (Costar, USA) in complete medium at a cell density of 1 × 10^6^/mL. They were then grown under 5% CO_2_ at 37 °C. For passaging, cells were digested with 0.25% trypsin at 90% confluency.

### FACS

Cultured cells at passage three (1 × 10^6^ cells/specimen) were harvested via trypsinization and incubated with the following fluorescent molecule-conjugated antibodies: anti-rat CD29-FITC (561796, BD Biosciences, USA, 1:200), CD29 isotype control (553960, BDBiosciences, USA, 1:200), anti-ratCD34-APC (ab213058, Abcam, UK, 1:200), CD34-APC isotype control (552893, BDBiosciences, USA, 1:200), anti-rat CD45-PE-cy5 (559135, BD Biosciences, 1:200), CD45-PE-cy5 isotype control (550618, BD Biosciences, 1:200), anti-rat CD44-PE (554869, BD Biosciences, 1:200), CD44-PE isotype control (554680, BD Biosciences, 1:200), anti-rat CD90-PE (554898, BD Biosciences, 1:200), and CD90-PE isotype control (550617, BD Biosciences, 1:200). CD29 was a hamster IgM, whereas the others were mouse IgGs. The cells were treated for 30 min in the dark and subsequently suspended in PBS (5 × 10^5^/mL) and analyzed using a FACSCanto II flow cytometer (BD Biosciences).

### Multilineage differentiation

Purified DPSCs at the 3^rd^ passage were differentiated into adipocytes and osteoblasts and hepatocytes in osteogenic and adipogenic differentiation medium, respectively, for 21 d. The osteogenic medium contained DMEM supplemented with 50 μg/mL ascorbic acid 2-phosphate, 0.1 µmol/L dexamethasone, and 10 mmol/L β-glycerol phosphate (Sigma-Aldrich) and 10% fetal bovine serum (GIBCO-BRL). The adipogenic medium contained DMEM supplemented with 0.1 µmol/L dexamethasone, 0.1 mmol/L indomethacin, 0.5 mmol/L 3-isobutyl-1-methylxanthine (Sigma-Aldrich) and 10% fetal bovine serum (GIBCO-BRL). DPSCs were differentiated into hepatocytes using the OriCell^TM^ Meschenchymal Stem Cell Hepatogenic Differetiation Medium (HUXMX-90101, Cyagen Biosciences Inc, USA) in strict accordance with Manufacturer’s instructions. Osteogenic, adipogenic and hepatogenic differentiation was verified by Alizarin Red (Sigma-Aldrich) staining, Oil Red O (Sigma-Aldrich) staining and periodic acid-Schiff (PAS; Sigma-Aldrich) staining, respectively. Hepatogenic differentiation was further confirmed by immunofluorescence staining using rabbit anti-albumin (Abcam; IgG; 1:300) according to the standard protocol.

### Lentiviral vector construction and transduction

Two lentiviral vectors (a blank lentiviral vector encoding only green fluorescent protein (GFP) and a hHGF-expressing lentiviral vector encoding GFP and hHGF) were purchased from Shanghai Genechem Company **(**GOSL49905**)**. The lentiviral transduction of DPSCs with a multiplicity of infection (MOI) of 20 was carried out for 72 h in 8 mg/mL polybrene (Millipore, USA). The transduction efficiency was evaluated by observation using a BX51 fluorescence microscope (Olympus, Japan).

### Rat model of cirrhosis and cell transplantation

To induce liver cirrhosis, 40% CCl_4_ in olive oil (0.2 mL/100 g body weight) was injected intraperitoneally into female Sprague-Dawley rats (200–250 g) in the cirrhosis model groups twice weekly for 12 weeks. Rats in the Control group received an intraperitoneal injection of the same volume of saline. For transplantation, on Week 12, wild-type, Lenti-GFP-transduced or Lenti-hHGF-transduced DPSCs (1 × 10^6^) in 0.5 mL of PBS were injected into the caudal veins of the rats in the cell transplantation groups using a Hamilton Syringe. Rats in the Vehicle group received an intravenous injection of the same volume of PBS.

### Serum assays

For the rat serum assays, blood was harvested from the abdominal aorta and transferred to coagulation-promoting vacuum tubes (MDSIN, China) to collect the serum. To evaluate the serum levels of albumin, alanine aminotransferase (ALT) and aspartate aminotransferase (AST), enzyme-linked immunosorbent assays (ELISAs) were performed using ELISA kits (Invitrogen) according to the manufacturer’s instructions.

### Histopathological analysis

After the rats were sacrificed, the right lobes of their livers were dissected and embedded in paraffin. Serial 4-µm sections of the right lobes of the livers were prepared. Routine hematoxylin & eosin (HE) and Masson’s trichrome staining were performed according to standard protocols. Fibrosis was evaluated using the Masson’s trichrome staining-based Laennec fibrosis scoring system^24^. In this system, hepatic fibrosis is scored from 0 to 6, with 0 indicating no definite fibrosis; 1, minimal fibrosis; 2, mild fibrosis; 3, moderate fibrosis; 4, mild, but definite cirrhosis; 5, moderate cirrhosis; and 6, severe cirrhosis. An immunofluorescence assay was conducted to evaluate the survival and differentiation of grafted cells. The sections were stained using mouse anti-prealbumin (transthyretin, TTR; Santa Cruz), rabbit anti-cytokeratin (CK) 18 (Abcam) or anti-albumin (Abcam) at a dilution of 1:300 (all these antibodies were IgGs), followed by incubation with Alexa 488-conjugated goat anti-rabbit IgG (R37116, Invitrogen, 1:400) and anti-mouse IgG (R28177, Invitrogen, 1:400). The stained sections were visualized, and images were captured using an IX83 confocal microscope (Olympus, Japan). The GFP^+^ cells in local area were counted, the precentage resident GFP^+^ cells of serial sections in total grafted cells were calculated and colocalization of GFP and TTR, CK18 or albumin was measured using the software Image-Pro Plus 6.0.

### Quantitative PCR

Total RNA was extracted from liver tissue using TRIzol reagent (HaiGene, Harbin, China). cDNA was then synthesized using the Golden 1st cDNA Synthesis Kit (HaiGene, Harbin, China), and real-time PCR was performed using Golden HS SYBR Green qPCR Mix (HaiGene, Harbin, China) with a MiniOpticon2 system (MJ). The reaction conditions were 95 °C for 15 min, followed by 40 cycles of 95 °C for 5 s and 60 °C for 30 s. The primers for the target genes (rat) were as follows: albumin (forward: 5′-AGACGGTGATGGGTGACTTC-3′, reverse: 5′-GCCTGAGATGGTTGTGATG TG -3′); TTR (forward: 5′-ACAGATGAGAAGTTCACGGAAGG-3′, reverse: 5′-CGATGGTGTAGTGGCGATGAC-3′); CK18 (forward: 5′-TCCGTGCCCAGTAT GAACAG-3′, reverse: 5′-AGTCCAGGTCAATCTCCAAGG-3′); and glyceraldehyde 3-phosphate dehydrogenase (GAPDH; forward: 5′-AACTCCCATTCTTCCACCTTT-3′, reverse: 5′-CTCTTGCTCTCAGTATCCT TG-3′). The mRNA expression of the target genes was normalized to GAPDH expression and calculated using the 2^−ΔΔCt^ method.

### Western blotting

Total protein was extracted using Super RIPA Lysis Buffer with PMSF (HaiGene, Harbin, China). Then, 30 µg of total protein was subjected to SDS-PAGE and transferred to a PVDF membrane (Millipore, USA). Target protein expression was detected using the following primary antibodies: rabbit anti-HGF (ab178359, Abcam, UK), rabbit anti-hHGF (ab24865, Abcam, UK), rabbit anti-rat CK18 (ab133263, Abcam, UK) or anti-rat albumin (ab207327, Abcam, UK), and mouse anti-TTR (SC-377517, Santa Cruz) (IgGs; 1:000). β-actin expression was used as a control using rabbit anti-β-actin (Sigma-Aldrich, USA, 1:000) The protein blots were visualized using horseradish peroxidase-conjugated goat anti-rabbit IgG (65–6120, Invitrogen, USA, 1:10,000), anti-mouse IgG (A28177, Invitrogen, USA, 1:10,000) and an enhanced chemiluminescence (ECL) system (BioRad, USA). Protein expression levels were measured using Image-Pro Plus 6.0 software. All of the antibodies used in this study were list in Table 1.

**Table 1:** List of antibodies used.

Antibody	Manufacturer	Source	Dilution (FACS)	Dilution (IF)	Dilution (WB)
Albumin	Ab207327, Abcam	Rabbit		1:300	1:1000
TTR	Sc-377517 Santa Cruz	mouse		1:300	1:1000
CK18	Ab133263 Abcam	Rabbit		1:300	1:1000
CD29-FITC	561796 BD Biosciences	Hamster	1:200		
CD29-FITC Isotype Control	553960 BD Biosciences	Hamster	1:200		
CD90-PE Isotype Control	554898 BD Biosciences	Mouse	1:200		
CD90-PE Isotype Control	550617 BD Biosciences	Mouse	1:200		
CD45-PE-cy5	559135 BD Biosciences	Mouse	1:200		
CD45-PE-cy5 Isotype Control	550618 BD Biosciences	Mouse	1:200		
CD44-PE	554869 BD Biosciences	Mouse	1:200		
CD44-PE Isotype Control	554680 BD Biosciences	Mouse	1:200		
CD34-APC	Ab213058 Abcam	Mouse	1:200		
CD34-APC Isotype Control	552893 BD Biosciences	Mouse	1:200		

### Statistical analysis

Statistical analyses were performed using Microsoft Excel and SPSS 23.0 software. The data are expressed as the mean ± standard deviation (SD) or the mean ± standard error of the mean (SEM). Statistical significance was determined using Student’s t test or analysis of variance (ANOVA), followed by Bonferroni’s post hoc multiple comparisons. *P* < *0.05* was considered to indicate statistical significance.

## Results

### Rat model of liver cirrhosis

**Figure 2:**
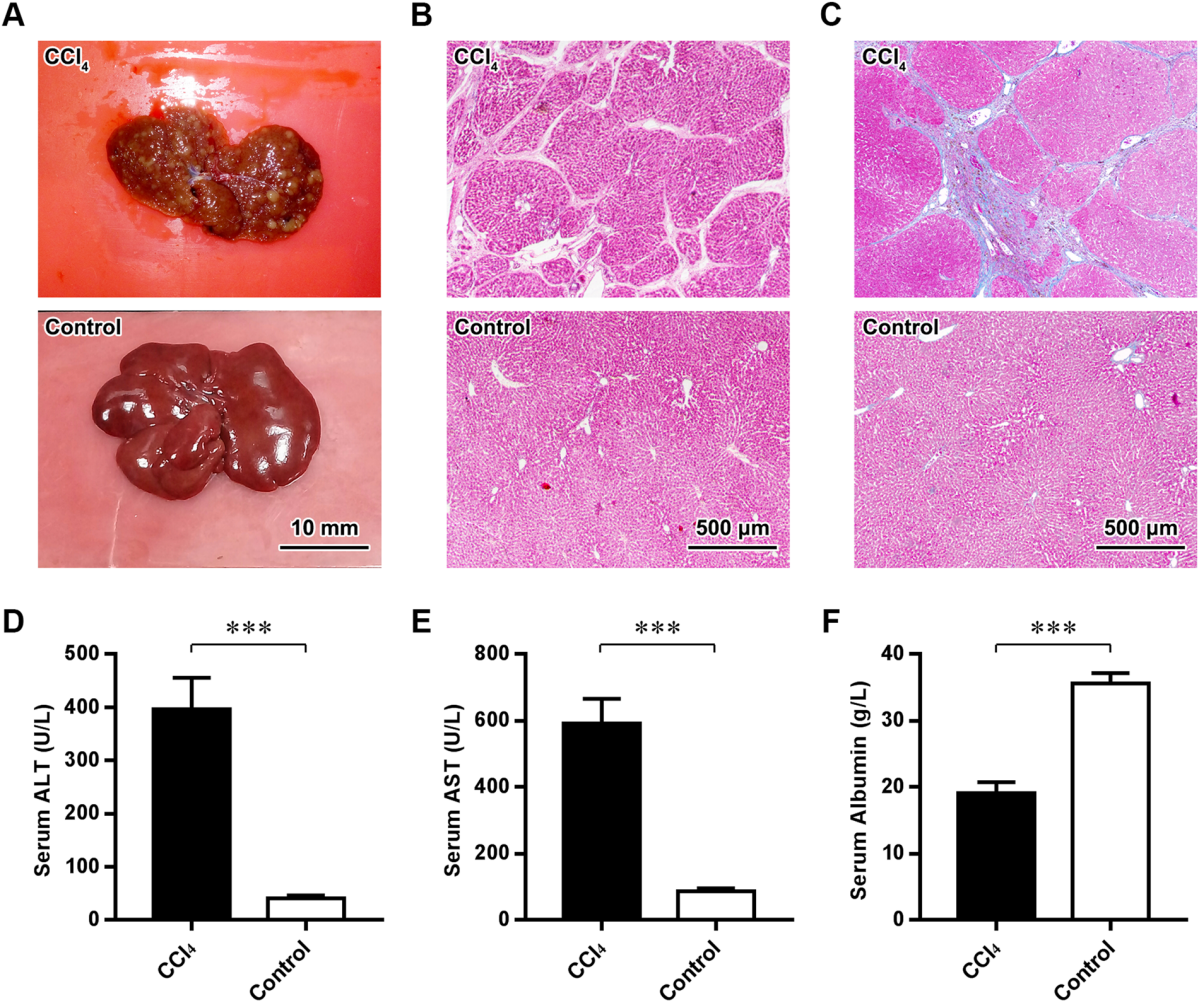
Rat model of liver cirrhosis. (**A**–**C**) Gross observations (**A**), HE staining (**B**) and Masson’s trichrome staining of the livers of rats in the CCl_4_ and Control groups. Scale bars (**A**) = 10 mm; (**B**,**C**) = 500 μm. (**D**–**F**) ELISA of the serum ALT, AST and albumin levels of rats in the CCl_4_ and Control groups. ***P < 0.001.

To recapitulate liver cirrhosis, rats were administered intraperitoneal injections of CCl_4_ for 12 weeks. The livers of CCl_4_-treated rats exhibited a hard texture and a rough surface with numerous nodules compared with those of control rats, which exhibited a soft texture and smooth surface (Fig. 2A). HE staining and Masson’s trichrome staining further revealed diffuse parenchymal regenerating nodules and fibrous septa in the livers of rats in the CCl_4_ group (Fig. 2B,C). To assess hepatic dysfunction, serum assays were performed. As shown in Fig. 2D–F, significant increases in the levels of serum alanine aminotransferase (ALT) and aspartate aminotransferase (AST) and a simultaneous decrease in serum albumin occurred after CCl_4_ treatment (all *P* values < 0.001). These results indicated that the major histopathological and serological changes of liver cirrhosis had emerged in CCl_4_-treated rats.

### Isolation and characterization of DPSCs

**Figure 3:**
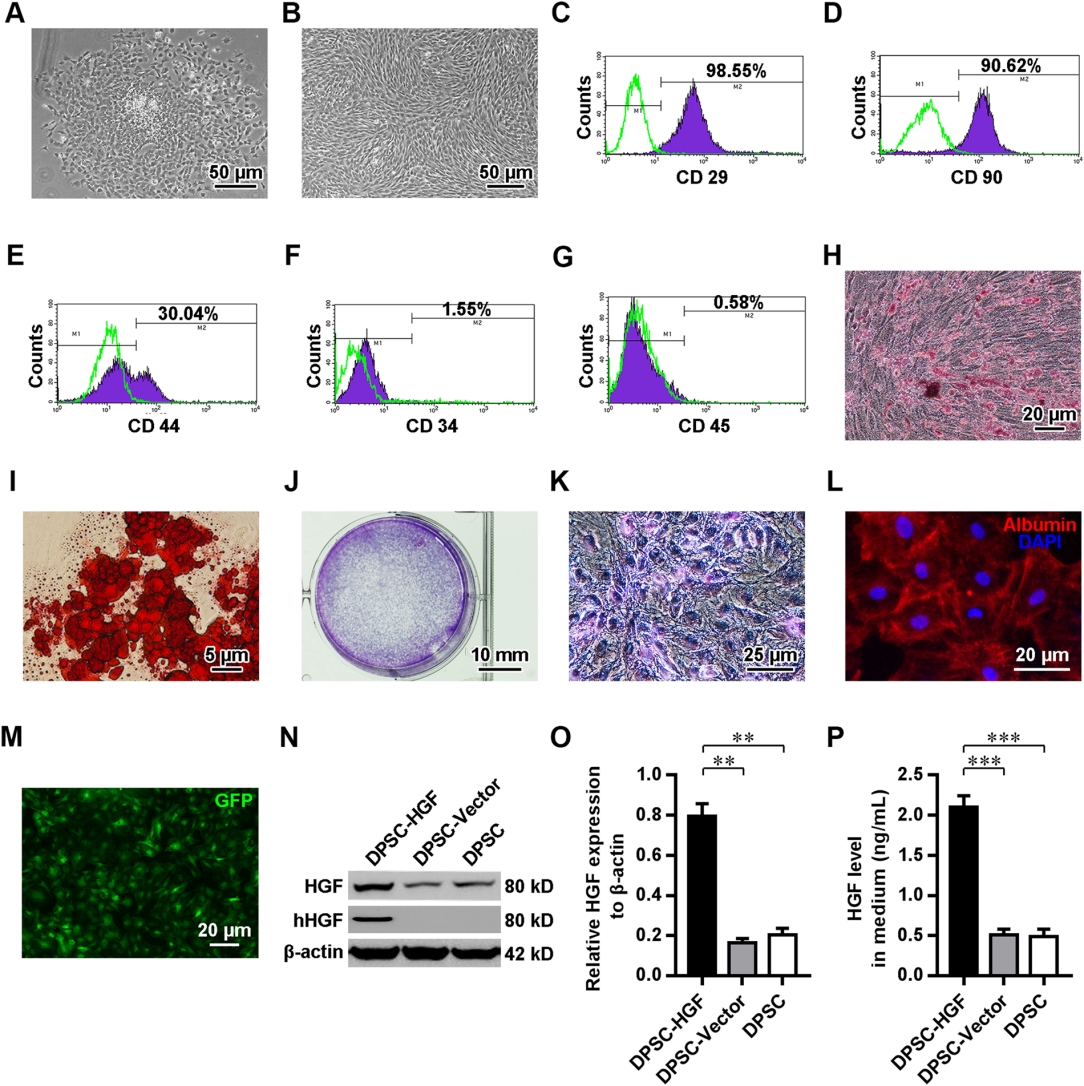
Isolation and characterization of DPSCs. (**A**,**B**) Phase-contrast images of primary DPSCs cultured for 48 h (**A**) and DPSCs at the 3^rd^ passage (**B**). (**C**–**G**) FACS analyses of the expression of CD29 (**C**), CD90 (**D**), CD44 (**E**), CD34 (**F**) and CD45 (**G**) in DPSCs. (**H**,**I**) Images of Alizarin Red (**C**) and Oil Red O staining (**D**) in DPSCs cultured in osteogenic and adipogenic differentiation medium for 21 d. (**J**–**K**) Gross (**J**) and phase contrast (**K**) images of PAS staining in DPSCs grown in hepatogenic differentiation medium for 17 d. (**L**) Fluorescence microscopic image of DPSCs grown in hepatogenic differentiation medium for 17 d, immunostained with anti-albumin (red) and counterstained with DAPI (blue). (**M**) Fluorescence microscopic image of GFP (green)-encoding lentiviral vector-transduced DPSCs. (**N**,**O**) Images from Western blot analyses for HGF and hHGF in DPSCs (**N**) and related quantitative analysis (**O**). (**P**) ELISA of HGF levels in the medium of DPSCs. Scale bars (**A**,**B**) = 50 μm; (**H**,**L**,**M**) = 20 μm; (**J**) = 5 μm; (**J**) = 10 mm; (**K**) = 25 μm. **P < 0.01; ***P < 0.001.

The primary dental pulp-derived cells displayed adherent growth and had formed multiple colonies 48 h after their initial plating (Fig. 3A). The cultured cells at the 3^rd^ passage showed a relatively uniform spindle-like morphology and a vortex-like distribution (Fig. 3B). To characterize the immunophenotypes of these dental pulp-derived cells, cells at the 3^rd^ passage were subjected to flow cytometry (Fig. 3C–G). Intriguingly, the vast majority of cultured dental pulp-derived cells expressed CD29 and CD90, which are markers of MSCs. Approximately 30% of the cells expressed CD44 (a homing cell adhesion molecule), whereas they rarely expressed CD34 (a hematopoietic progenitor marker) or CD45 (leukocyte common antigen). To evaluate the multipotency of these cultured dental pulp-derived cells, osteogenic, adipogenic and hepatogenic differentiation was induced. When grown in osteogenic and adipogenic differentiation media, the cultured cells developed into osteoblasts (Fig. 3H) or adipocytes (Fig. 3I), as shown using Alizarin Red staining for mineral deposits and Oil Red O staining for lipid vacuoles, respectively. Futhermore, when exposed to the hepatogenic differentiation media, these dental pulp-derived cells granually gained irregular polygonal and paving stone-like morphology which resembled that of primary hepatocyte (Fig. S1A–F). The hepatocyte-like glycogen accumulation (Figs 3J,K and S1G) and emerging expression of hepatocyte-produced albumin (Fig. 3L) in these dental pulp-derived cells were further revealed by PAS staining and immunostaining respectively. These data indicated that the dental pulp-derived cells with MSC-like immunophenotypes and osteogenic, adipogenic and hepatogenic multipotency were DPSCs.

### HGF overexpression promotes the hepatocyte-like differentiation of grafted DPSCs

To investigate the effects of HGF on the determination of DPSC fate, rat DPSCs at the 3^rd^ passage were transduced with the human HGF (hHGF)-lentiviral vector (DPSC-HGF) or a blank lentiviral vector (DPSC-Vector). Overexpression of HGF in hHGF-lentiviral vector-transduced cells was confirmed through the expression of the reporter gene (green fluorescence protein, GFP; Fig. 3M,N) as well as hHFG and the over 4-fold increase in total HGF levels in both the cells and medium (Fig. 3O,P), relative to blank lentiviral vector-transduced and wild-type (DPSC) DPSCs (both *P* values < 0.001).

**Figure 4:**
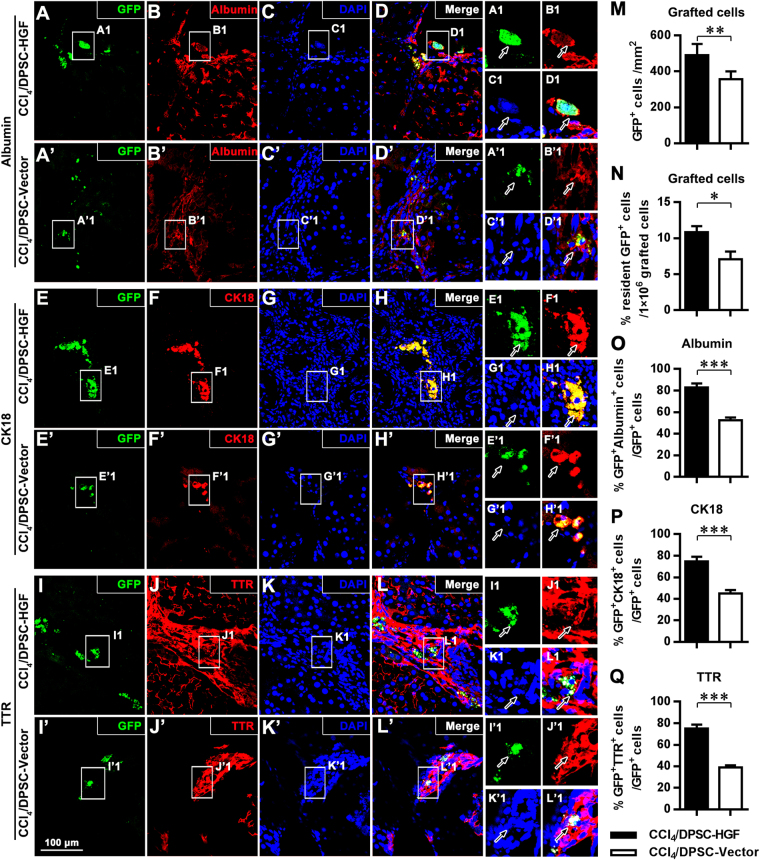
HGF overexpression promotes the hepatocyte-like differentiation of grafted DPSCs. (**A**–**L**,**A’**–**L’**) Fluorescence microscopy images of hepatic tissue immunostained with anti-GFP (**A**,**A’**,**E**,**E’**,**I**,**I’**), anti-albumin (**B**,**B’**), anti-CK18 (**F**,**F’**), and anti-TTR (**J**,**J’**) and counterstained with DAPI (**C**,**C’**,**G**,**G’**,**K**,**K’**) and related merged fluorescence microscopy images (**D**,**D’**,**H**,**H’**,**L**,**L’**) in CCl_4_/DPSC-HGF (**A**–**L**) and CCl_4_/DPSC-HGF groups (**A’**–**L’**). A1-L1 and A’1-L’1 show magnified images of the outlined areas from the corresponding panels. (M-Q) Quantitative analyses of the number of GFP^+^ graft-derived cells (**M**), the percentage of resident GFP^+^ cells in total 1 × 10^6^ grafted cells (**N**) and the percentages of albumin^+^ (**O**), CK18^+^ (**P**) and TTR^+^ (**Q**) cells in the GFP^+^ graft-derived cell population. Scale bar (**A**–**L**,**A’**–**L’**) = 100 μm. **P < 0.01; ***P < 0.001.

The lentiviral vector-transduced DPSCs were subsequently transplanted into rats with cirrhosis through intravenous injection. Immunofluorescence staining and confocal microscopy analysis performed 6 weeks after transplantation showed that increased amounts of GFP-positive graft-derived cells survived, migrated to and resided in the host liver tissue areas in the DPSC-HGF group compared with those in the DPSC-Vector group (*P* < 0.01; Fig. 4A–L,A’–L’,M). Further image analyses of serial sections verified that higher percentages of GFP-positive cells resident in host liver tissue in total grafted cells existed in DPSC-HGF group (10.84% ± 0.84%) rather than DPSC-Vector group (7.08% ± 1.07%; *P* < 0.05; Fig. 4N). Furthermore, the GFP fluorescence of the grafted DPSCs colocalized with the immunofluorescence of the single-layer epithelial marker CK18 as well as the hepatocyte-synthesized plasma proteins albumin and TTR, which suggested that the grafted DPSCs with hepatogenic potency confirmed *in vitro* (Figs S1 and 3J–L), had differentiated into hepatocyte-like cells in host liver tissue with cirrhosis pathology. Quantitative analyses further indicated that the grafted DPSCs with the elevated expression of HGF gave rise to higher frequencies of GFP^+^albumin^+^ (*P* < 0.001; Fig. 4O), GFP^+^CK18^+^ (*P* < 0.001; Fig. 4P) or GFP^+^TTR^+^ (*P* < 0.001; Fig. 4Q) graft-derived hepatocyte-like cells than with the blank lentiviral vector-transduced DPSCs. These results verified that the hepatocyte-like differentiation of grafted DPSCs in host liver tissue with cirrhosis could be significantly enhanced by HGF overexpression.

### HGF overexpression enhances the grafted DPSC-mediated amelioration of cirrhosis in rats

**Figure 5:**
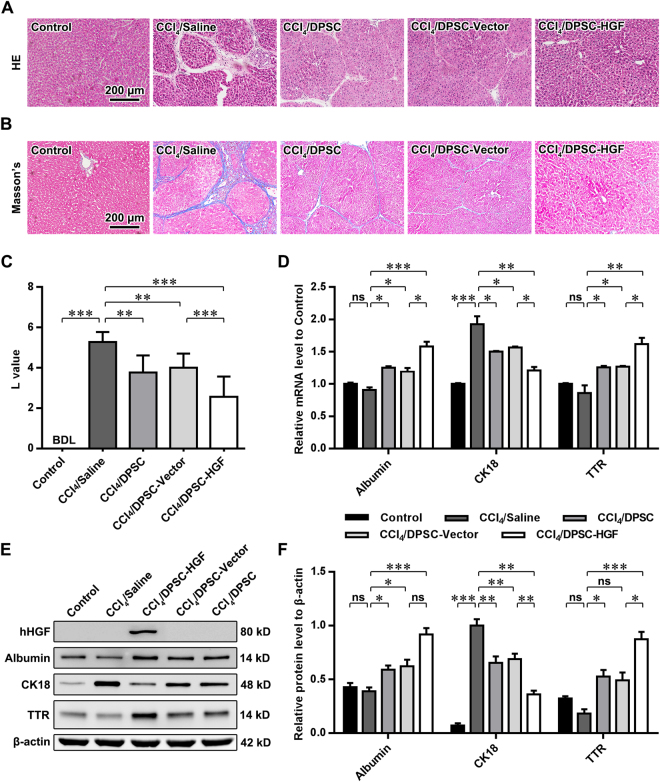
HGF overexpression in grafted DPSCs enhances the amelioration of cirrhosis in a rat cirrhosis model. (**A**,**B**) Images of the HE staining (**A**) and Masson’s trichrome staining (**B**) of the liver tissue of rats. (**C**) Quantitative analysis of the Laennec fibrosis scoring for the liver tissue of rats. (**D**) Quantitative analyses of qPCR assays for the mRNA levels of albumin, CK18 and TTR in the liver tissue of rats. (**E**,**F**) Images of Western blot analyses for the protein expression of hHGF, albumin, CK18 and TTR in the liver tissue of rats (**E**) and the related quantitative analysis (**F**). Scale bars (**A**,**B**) = 200 μm. ns, nonsignificant; **P < 0.05; **P < 0.01; ***P < 0.001.

To further study the impacts of HGF on the repair potential of grafted DPSCs in cirrhosis, histopathological analyses were conducted on healthy rats (Control), sham-treated rats with cirrhosis (CCl_4_/Saline) and rats receiving grafts of wild-type DPSCs (DPSC), blank lentiviral vector (CCl_4_/DPSC-Vector)-transduced DPSCs or hHGF-lentiviral vector (CCl_4_/DPSC-HGF)-transduced DPSCs for 6 weeks. As shown through HE staining (Fig. 5A), the formation of regenerative nodules and fibrous septa was notably attenuated in the livers of rats with cirrhosis after DPSC grafting. Masson’s trichrome staining and subsequent image analysis (Fig. 5B,C) further revealed that cirrhosis-specific fibrosis was significantly alleviated in the livers of model rats receiving grafts of wild-type, blank lentiviral vector or hHGF-lentiviral vector-transduced DPSCs (all *P* values < 0.01 vs. CCl_4_/Saline). Compared with rats in the CCl_4_/DPSC-Vector group, the rats in the CCl_4_/DPSC-HGF group exhibited a better recovery from hepatic fibrous deposition (*P* < 0.001).

Molecular biological analyses were utilized to determine the levels of markers of mature and functional hepatocytes in liver tissues. Interestingly, qPCR (Fig. 5D) revealed that the sham-treated rats with cirrhosis displayed unaltered albumin and TTR mRNA levels (both *P* values > 0.05) and dramatically increased CK18 mRNA levels (*P* < 0.001) compared with the healthy Controls, whereas all the rats in the DPSC-treated groups exhibited increased albumin and TTR levels and restored CK18 transcription (all *P* values < 0.05). Furthermore, rats treated with hHGF-lentiviral vector-transduced DPSCs showed comparatively elevated albumin and TTR mRNA levels and recovered CK18 transcription relative to those of rats treated with blank lentiviral vector-transduced DPSCs (all *P* values < 0.05). Concurrent with the *in vitro* observations, the specific expression of human HGF in the liver tissue of rats subjected to grafting of hHGF-lentiviral vector-transduced DPSCs was detected by Western blotting (Fig. 5E,F). Consistent with the qPCR data, Western blotting (Fig. 5E) indicated no significant changes in albumin or TTR expression (both *P* values > 0.05) in the liver tissue of rats with cirrhosis, whereas albumin and TTR levels were increased in rats treated with DPSCs (both *P* values < 0.05). Additionally, TTR expression was further up-regulated in the CCl_4_/DPSC-HGF group (*P* < 0.05). Furthermore, the aberrant rise in CK18 expression in rats with cirrhosis was reversed after treatment using DPSCs expressing HGF at normal levels (both *P* values < 0.01) and was further restored by the grafting of HGF-overexpressing DPSCs (*P* < 0.01). These data indicate that the regenerative and repair capacity of grafted DPSCs in cirrhosis-related pathological injury was enhanced by HGF overexpression.

### HGF overexpression in grafted DPSCs enhances the hepatic functional recovery of rats with cirrhosis

**Figure 6:**
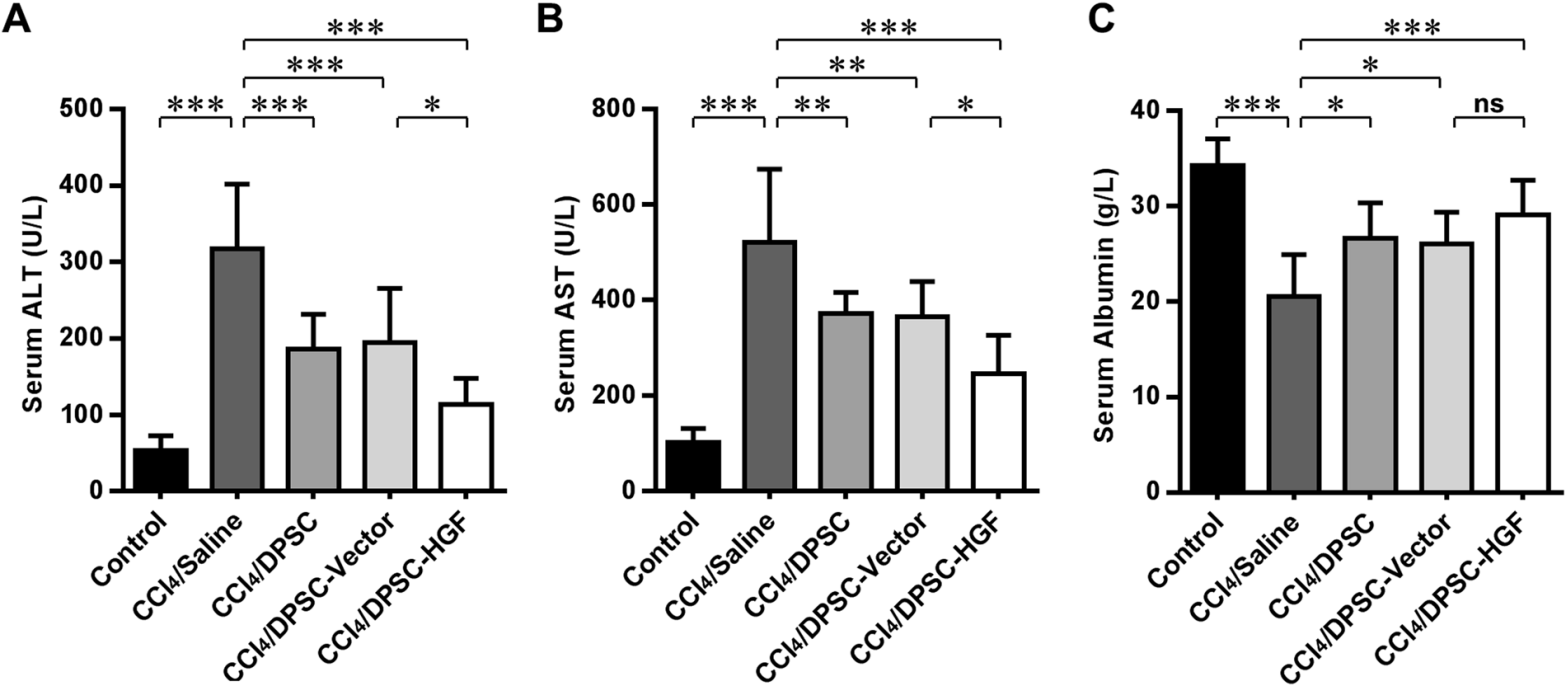
HGF overexpression in grafted DPSCs enhances the hepatic functional recovery of rats with cirrhosis. (**A**–**C**) ELISAs of the serum ALT (**A**), AST (**B**) and albumin (**C**) levels of the rats in each group. ns, nonsignificant; **P < 0.05; **P < 0.01; ***P < 0.001.

To further evaluate the effect of HGF on the protective efficacy of DPSCs against hepatic functional impairment, serum assays were performed. As shown by the ELISA results (Fig. 6), the increases in the serum levels of the enzymes ALT and AST released by injured hepatocytes in rats with cirrhosis (both *P* values < 0.001 vs. Control) were alleviated 6 weeks after the grafting of DPSCs with physiological HGF levels (all *P* values < 0.01) and recovered more significantly in rats treated with DPSCs overexpressing HGF (both *P* values < 0.05). Intriguingly, inconsistent with the results of Western blotting, the evident decrease in the level of serum albumin in cirrhosis model rats (*P* < 0.001 vs. Control) was recovered to equivalent levels via the grafting of physiological HGF-expressing and HGF-overexpressing DPSCs (all *P* values < 0.05 vs. CCl_4_/saline). These data suggest that the grafted DPSC-induced hepatic functional recovery could be further improved through elevated HGF expression.

## Discussion

In this study, we investigated the impacts of hHGF overexpression on the *in vivo* biological behaviors and the therapeutic efficacy of DPSCs for the treatment of liver cirrhosis. Purified multipotent rat dental pulp-derived mesenchymal stem cells were characterized for their immunophenotype, transduced with hHGF-lentiviral vectors to elevate the expression and secretion of HGF, and transplanted into rats with confirmed CCl_4_-induced cirrhosis. The HGF-overexpressing DPSCs showed significantly increased survival and hepatocyte-like differentiation in host liver tissue and dramatically enhanced the repair potential for cirrhosis pathology and impaired liver function compared with DPSCs expressing HGF at physiological levels.

CCl_4_-induced hepatic injury in rats is widely used as an induced animal model of liver fibrosis because of its high similarity to the morphological and functional alterations observed in human cirrhosis^25–27^. In this study, CCl_4_ was administered to rats twice weekly at an appropriate dosage, determined in preliminary experiments, for approximately 3 months, rather than administering a relatively high dose for a short term, to ensure the survival of the experimental animals and the emergence of identifiable pathology. As shown through histopathological and serological analyses, cirrhosis-specific formation of hepatic regenerating nodules and fibrous septa, increased serum levels of aminotransferases indicative of hepatocyte injury and a decreased level of hepatocyte-synthesized serum albumin occurred in the CCl_4_-treated rats, verifying that liver cirrhosis was well recapitulated in the rats.

Cirrhosis, the terminal stage of hepatic diseases, is prone to develop into hepatocyte carcinoma and hepatic failure, which is incurable in clinical practice, except through liver transplantation. Fortunately, in preclinical studies, interventions such as MSC graft-based regenerative medicine therapies, involving minimally invasive procedures and few complications, have been demonstrated to cause hepatic fibrosis to regress and to improve clinical outcomes, even in advanced stages of cirrhosis^28,29^. Among the various lineages of MSCs, embryonic ectomesenchyme tissue-derived uncommitted DPSCs (the target cell type in this study), which are capable of homing, self-renewal and differentiation in injured tissue, are considered one of the therapies with the most potential for use in the treatment of tissue injury. These cells display obvious advantages over other types of stem cells, including ease of accessibility for autografts, minimal immune rejection and tolerance to stress, including long-term cryopreservation^12,24,30^. The primary rat DPSCs isolated and purified in this study were found to express MSC surface markers (CD90 and CD29) and show some expression of the homing cell adhesion molecule CD44, but they rarely expressed hematopoietic lineage-related molecules (CD34 and CD45). These cells also exhibited osteogenic and adipogenic potency (Fig. 3). All these characteristics were in accord with the characteristics of DPSCs identified in previous studies^31,32^. With regard to hepatic regeneration, differentiation of DPSCs into functional hepatocyte-like cells that express hepatocyte markers, such as albumin, CK18 and TTR, and are capable of repairing liver injury *in vitro* and *in vivo* has been observed in several independent studies^14,33–36^ and was further confirmed by our work (Figs S1 and 3, 4 and 5). However, the key regulatory mechanisms underlying hepatocyte-like commitment and the hepatic regenerative capacity of DPSCs remain elusive.

HGF is a mesenchymal cell-secreted cytokine that extensively regulates the growth, motility and morphogenesis of a variety of cell types, especially hepatocytes^15,16^. Intriguingly, mesenchymal cell-derived HGF has been demonstrated to stimulate the differentiation of bone marrow-resident MSCs into hepatocyte-like cells^20,21^. Accordingly, our results showed that the intravenous transplantation of HGF-overexpressing DPSCs (dental pulp-derived MSCs) gave rise to higher frequencies of albumin-positive, CK18-positive and TTR-positive hepatocyte-like cells than did DPSCs expressing HGF at physiological levels (Fig. 4), which further clarified the general hepatogenic differentiation-inducing capacity of HGF in MSCs. Moreover, we found more graft-derived GFP-labeled cells in the liver tissue of animals receiving grafts of DPSCs with elevated HGF expression, suggesting possible positive modulation of the homing and survival of grafted DPSCs by HGF.

To evaluate the repair potential of HGF-overexpressing DPSCs for cirrhosis, the morphological, molecular and functional recovery of the rat livers was tested 6 weeks after transplantation, rather than within 4 weeks as in previous studies^37,38^. The longer observation period ensures the effective functioning of DPSCs as well as hepatic regeneration and increases the reliability and precision of the research results. As shown by histopathological tests, the DPSC grafting-facilitated reconstruction of the liver architecture and reversal of hepatic fibrosis were further reinforced by HGF overexpression (Fig. 5). The benefits of HGF-overexpressing DPSC grafting might result from both the HGF-accelerated hepatogenic differentiation of DPSCs and the specific expression and secretion of human HGF itself. The ectopic secretion of a high dose of hHGF far above intrinsic HGF secretion levels may lead to the activation of resident hepatic progenitor cells, which have been implicated in hepatic regeneration^39^, and/or the obvious anti-fibrogenic activities of HGF^17,18^. Additionally, the DPSC graft-promoted hepatocyte regeneration, enhanced by the overexpression of HGF in transplanted cells, was verified by the restoration of the mRNA and/or protein expression of albumin, TTR and CK18 (Fig. 5D–F), as reduced hepatic levels of albumin and TTR (hepatocyte-synthesized plasma proteins) and aberrantly increased expression of CK18 (single-layer epithelial Type I intermediate protein) associated with hepatogenic dedifferentiation are well-established indicators of cirrhosis-related hepatocyte injury^40–43^. Interestingly, as revealed by serum assays, the HGF-overexpressing DPSC-treated rats gained further recovery of the serum levels of aminotransferases, but not albumin, compared with the physiological HGF-expressing DPSC-treated animals. This result was consistent with the Western blot data for liver tissue and might suggest the heterogeneous restoration of hepatic function.

In summary, our results demonstrated that MSC-specific-marker-expressing multipotent DPSCs transduced with a hHGF-lentiviral vector showed elevated HGF expression and secretion of HGF *in vitro*. These cells also exhibited markedly increased survival and underwent hepatogenic differentiation in host liver tissue, significantly enhancing their therapeutic efficacy in liver cirrhosis. Our study provides a novel method for the treatment of liver cirrhosis in clinical practice.

## Electronic supplementary material

Figure-S1
