# Supplementary material for: Therapeutic effects of hepatocyte growth factor-overexpressing dental pulp stem cells on liver cirrhosis in a rat model
Source: Sci Rep. 2017 Nov 17;7:15812. doi: 10.1038/s41598-017-14995-5 (PMC5693919; doi:10.1038/s41598-017-14995-5)
Supplement: Supplementary file 1 — Figure-S1 [file 41598_2017_14995_MOESM1_ESM.pdf]

Therapeutic effects of hepatocyte growth factor-overexpressing dental pulp stem cells on liver cirrhosis in a rat model

Xiao-fang Cao[1#], Shi-zhu Jin[2#], Liang Sun[3], Yuan-bo Zhan[4], Feng Lin[4], Ying Li[4], Ying-lian Zhou[5], Xiu-mei Wang[1], Li Gao[1] and Bin Zhang[4,6※]

1. Department of Dentistry, Second Affiliated Hospital of Harbin Medical University, Harbin, 150086, Heilongjiang, China

2. Department of Gastrointestinal and Hepatology, Second Affiliated Hospital of Harbin Medical University, Harbin, 150086, Heilongjiang, China

3. Department of Human Anatomy, Harbin Medical University, Harbin 150081, Heilongjiang, China.

4. Institute of Hard Tissue Development and Regeneration, Second Affiliated Hospital of Harbin Medical University, Harbin 150001, Heilongjiang, China

5 Department of neurology, The Second Affiliated Hospital of Harbin Medical University

6. Heilongjiang Academy of Medical Sciences, Harbin 150001, Heilongjiang, China

[#]*Xiao-fang CAO and Shi-zhu Jin* equally contributed to the manuscript.

[※]Corresponding author: Institute of Hard Tissue Development and Regeneration, Second Affiliated Hospital of Harbin Medical University, 246 Xue Fu Road, Harbin 150001, China

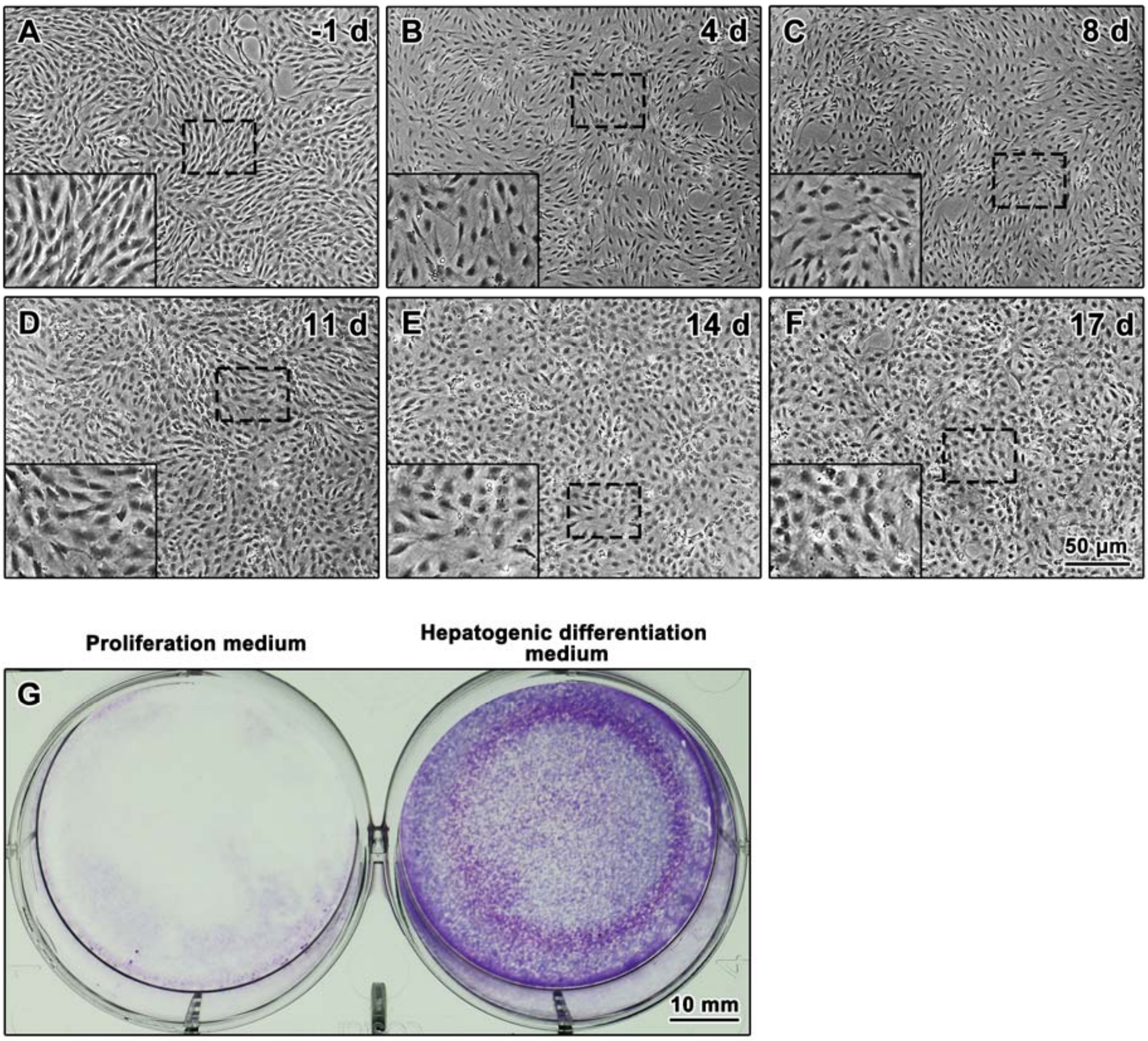

Figure-S1 Differentiation of DPSCs grown in hepatogenic differentiation medium. (A-F) Phase contrast images of DPSCs 1 day before and 4, 8,11,14, 17 days after culturing in hepatogenic differentiation medium. (G) Gross images of PAS staining in DPSCs grown in proliferation medium (left) and hepatogenic differentiation medium (right) for 20 d. Scale bar (A-F) =50 μm; (G)=10 mm.
